# Retraction Note: Middle east pain syndrome is a pollution-induced new disease mimicking rheumatoid arthritis

**DOI:** 10.1038/s41598-024-66447-6

**Published:** 2024-07-08

**Authors:** Adel A. Elbeialy, Abdlnby M. Bauomi, Basma M. Elnaggar, Hala M. Elzomor

**Affiliations:** 1 Rheumatology Department, Al-Azhar University Faculty of Medicine for Girls, 74 Ali Amin Street, Nasr City, Cairo 11727 Egypt; 2 Radiology Department, Al-Azhar Faculty of Medicine, Cairo, Egypt

Retraction of: *Scientific Reports* 10.1038/s41598-021-01698-1, published online 15 November 2021

Editors have retracted this Article.

After publication of this study, concerns were raised that:

- The data to support a correlation between cadmium/lead pollution and vitamin D3 deficiency, the presumed cause for the Middle East Pain Syndrome, is not presented;
- The absence of follow up studies and lack of consideration of alternative explanations of observed symptoms means it cannot be conclusively proven that the Middle East Pain Syndrome is a new disease;
- The findings of erosions are not backed by imaging data from MRI and CT Scans;
- The radiograph in Figure 2 is from a patient not included in this study.

Post-publication peer review by an expert has confirmed the validity of these concerns. The Editors therefore no longer have confidence in the conclusions of this study.

Adel A. Elbeialy and Abdlnby M. Bauomi disagree with this retraction. Basma M. Elnaggar and Hala M. Elzomor did not respond to correspondence from the Editors about this retraction.

